# Effect of mandibular position achieved using an oral appliance on genioglossus activity in healthy adults during sleep

**DOI:** 10.1186/s13005-019-0210-z

**Published:** 2019-11-04

**Authors:** Michikazu Matsuda, Toru Ogawa, Ratri M. Sitalaksmi, Makiko Miyashita, Toshimi Ito, Keiichi Sasaki

**Affiliations:** 1 Division of Advanced Prosthetic Dentistry, Tohoku University Graduate School of Dentistry, 4-1 Seiryo-machi, Aoba-ku, Sendai, Miyagi 980-8575 Japan; 2 Department of Prosthodontics, Faculty of Dental Medicine, Universitas Airlangga, Surabaya, Indonesia

**Keywords:** Obstructive sleep apnea, Sleep, Oral appliance, Mandibular position

## Abstract

**Background:**

Oral appliances (OAs) are generally designed to displace the mandible anteriorly and downward, to increase the airway patency. The present study aimed to examine the relationship between genioglossus (GG) muscle activity and mandibular position, considering both anterior and vertical displacements during sleep.

**Methods:**

Seven healthy male adults aged 29.4 ± 1.99 years were evaluated. Maxillary and mandibular OAs were fabricated from 2-mm-thick resin plates with pressure-welding. The activity of the left GG was recorded using two silver ball electrodes attached to the lingual edge of the mandibular OA. Respiratory status and right masseter muscle activity were measured by an airflow sensor and surface electrodes, respectively. Electroencephalography was used to determine the sleep status. Stage 2 (the second stage of sleep) was defined as the state of sleeping. Four test conditions with different mandibular positions (0 and 50% anterior protrusion) and bite openings (4 mm and 12 mm) were examined.

**Results:**

GG activity in SL4A (4 mm bite opening, 50% protrusion during sleep) and SL12 (12 mm bite opening, 0% protrusion during sleep) were significantly higher than that in SL4 (4 mm bite opening, 0% protrusion during sleep). Respiratory volume did not significantly differ between all test conditions.

**Conclusion:**

GG activity is influenced not only by anterior protrusion of the mandible but also by vertical displacement during sleep. Thus, when determining the effectiveness of intraoral appliances in the treatment of obstructive sleep apnea, both protrusion and the size of the mandibular opening should be evaluated and taken into account.

## Background

Obstructive sleep apnea syndrome (OSAS) is a life-threatening condition characterized by repeated pharyngeal airway collapse during sleep; affected subjects have a narrower pharyngeal airway than normal subjects. A study that evaluated the activity of the genioglossus (GG) muscle during sleep showed that subjects with OSAS had significantly greater activity reductions than normal subjects [1–3].

One of the treatments used for OSAS is the application of oral appliances (OAs); these are generally designed to increase the airway patency of patient by preventing the collapse of the mandible and correcting the tongue position. The most common OA type is that which holds the mandible in a more anterior position to enable correction of the anatomical balance, especially in the upper airways [4–8]. A previous study suggested that greater mandibular protrusion could decrease OSAS events, although the role of the vertical opening is still controversial and needs further investigation regarding the effects of a gradual increase in mandibular advancement in patients with OSAS [9]. Although OAs can have adverse effects [5, 6, 10], the benefits of using OAs outweigh the risks, especially when considering the life-threatening potential of OSAS. Therefore, patients should remain compliant with OA therapy unless it is replaced with other methods such as continuous positive airway pressure (CPAP) or upper airway surgery [5, 6].

Obesity is a precipitating factor for many diseases, including OSA and diabetes mellitus (DM), conditions that are interconnected. Those patients are likely to experience worse OSA symptoms. Continuous positive airway pressure is the standard treatment for those OSA patients, although OA therapy and weight loss through dietary and lifestyle modifications are important to the holistic management of such patients.

The GG muscle is a major contributor to preventing upper airways occlusion. Conversely, when under-stimulated, it plays a central role in apnea generation [11]. The respiratory-related activity of the GG is reportedly increased by anterior displacement of the mandible [12]; however, few studies have evaluated the changes in GG activity related to vertical displacement of the mandible. Physiological evidence suggests optimized treatment outcome when the bite opening (BO) is minimized [7]. A recent study investigating the relationship between GG activity and BO found that awake healthy subjects had significantly increased GG activity when the BO was 12 mm compared with 4 mm [13].

The present study aimed to examine the relationship between GG activity and mandibular position (both anterior and vertical displacements) during sleep in healthy adults. The null hypothesis was that the BO and anterior mandibular displacement would not affect GG activity, respiration rate, and breathing time during sleep.

## Material and methods

### Subjects

This research involving human participants was conducted in accordance with the ethical standards of the Ethical Committee of Tohoku University Graduate School of Dentistry (number: 23–4). The present study was carried out on seven healthy male volunteers with a mean ± standard deviation age and body mass index of 29.4 ± 1.99 years and 22.8 ± 2.56 kg/m^2^, respectively. Subjects were excluded if they had a respiratory disorder or infection, were taking medication that affected muscular activity, or had severe orofacial skeletal disharmony. Informed consent was obtained from each subject before study commencement.

### Experimental oral appliances

**Fig. 1:**
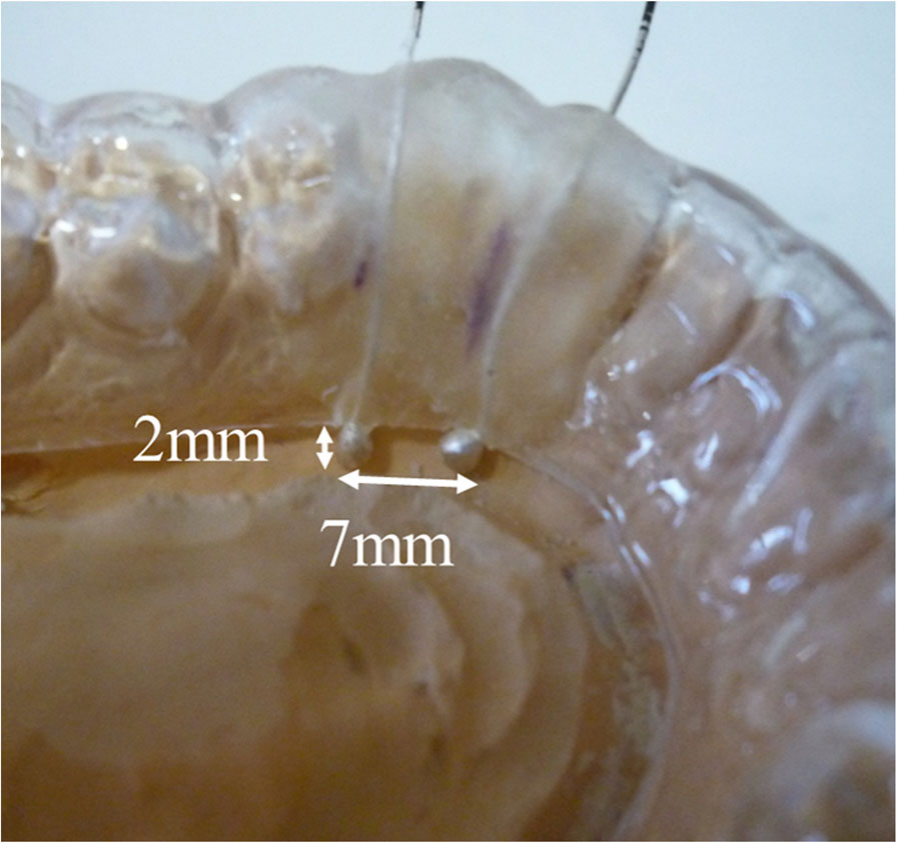
Photograph shows the experimental oral appliance with properly mounted electrodes used in the present study. Genioglossus activity was recorded using two silver ball electrodes attached to the lingual edge of the mandibular oral appliance

Maxillary and mandibular OAs were made from the dental cast of each subject. Before the impression of the mandible was created, border molding with an individual tray was performed using a modeling compound on the lingual side. In anterior region of the floor of the mouth, the most superiorly placed muscle is the GG; we have to reach the stability of an OA and to achieve adequate degree of tongue freedom and tissue reflection an accurate border molding in the vestibular spaces is a prime requisite [14]. The mandibular impression was taken using silicone impression pastes (Exafine regular and injection, GC, Tokyo, Japan), considering tongue mobility for the design of the lingual flange. Maxillary and mandibular OAs for each subject were fabricated from 2 mm-thick resin plates with pressure-welding (Erkodur, Erkodent Erich Kopp GmbH, Pfalzgrafenweiler, Germany), involving all the existing teeth in the jaw (Fig. 1).

### EMG recording

The left GG activity was recorded using two custom-made silver ball electrodes (2 mm diameter), which were incorporated in the lingual flange of the experimental OAs using a self-curing resin (UNIFAST II-clear, GC). The electrodes were positioned at the border of the lingual flange between the distal left lateral incisor and canine, with a distance of 7 mm between electrodes (Fig. 1). The position of the GG was confirmed as previously described to enable the monitoring of GG activity during respiration [13]. In this study, because we assumed that respiratory-related GG muscle activity is symmetric in normal subjects, we recorded only left GG muscle activity.

**Fig. 2:**
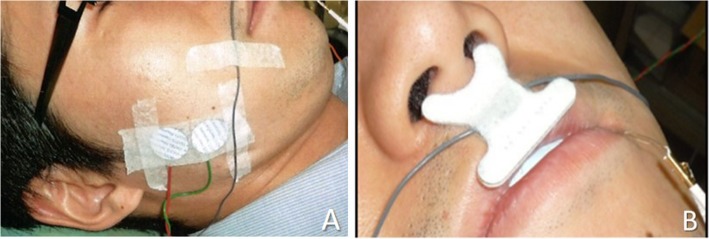
Photographs show the electrodes and airflow sensor used in the present study. **a**. Electrode placement on the right masseter muscle, with 15 mm between electrodes. **b**. Disposable airflow sensor placed in the nostrils to record the respiratory airflow

Masseter muscle activity was recorded using disposable surface electrodes (VIASYS electrode, Nicolet, WI, USA). The operator stood behind the subject and palpated the masseter muscles bilaterally while the patient gritted his teeth in the habitual occlusion position. Two electrodes were placed 3 cm from a gonial angle along the line that passed through the point from the lateral canthus of the eye [1]. The electrodes were attached to the center of the right masseter, with a distance of 15 mm between electrodes (Fig. 2a). The sampling frequency of the EMG recording was set at 1500 Hz.

Before the recording sessions, the masseter muscle and GG activity during maximum voluntary clenching and maximum tongue protrusion were recorded with the patient in an upright position to enable standardized evaluation of EMG data.

In every subject, measurements were recorded for 15 min after setting up the OAs (until the subject entered stage 2 of sleep). The airflow, and GG and masseter muscle activity were recorded simultaneously. Respiratory activity was recorded using a disposable airflow sensor (Dymedix Adult Airflow Sensors, Dymedix Corporation, MN, USA) (Fig. 2b).

### Electroencephalography (EEG)

**Fig. 3:**
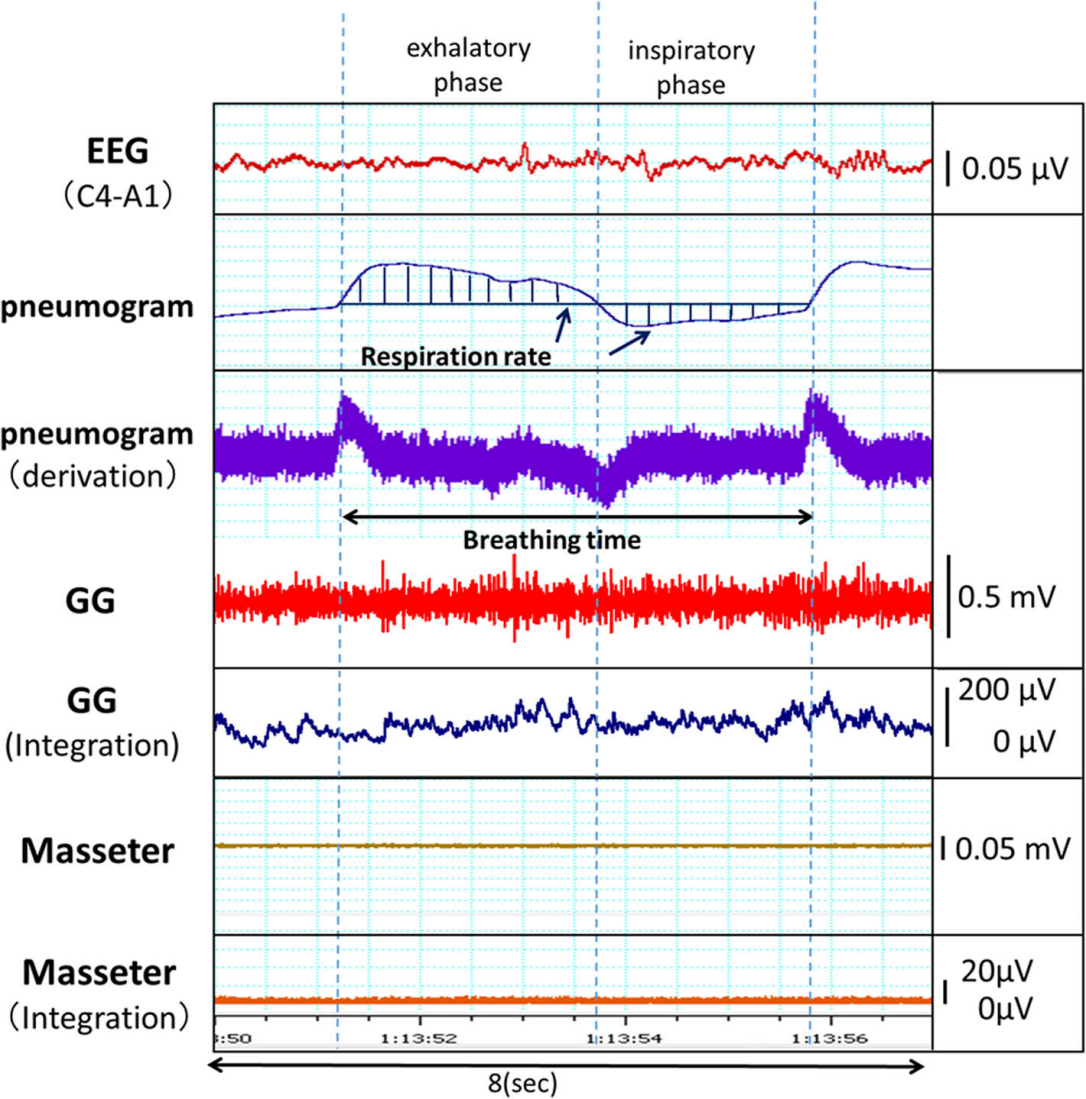
Examples of an electroencephalographic recording, pneumographic recording, and EMG recording

Five electrodes were positioned in accordance with the standard criteria of the 10–20 system of electrode placement proposed by the International EEG Society [15]; the electrodes were positioned at the top of the head on the right and left sides, at the occiput, and the earlobe. The sampling frequency was set at 200 Hz. EEG was recorded to determine the sleep status. In the present study, the state of sleeping was defined as stage 2 (the second stage of non-rapid eye movement sleep); stage 2 lasts approximately 10 to 25 min in the initial cycle, and subjects require stronger stimuli to invoke awakening in this stage than in stage 1 (Fig. 3) [16]. Subjects were considered to be in stage 2 when there was the appearance of a sleep spindle. Inspiration and exhalation were distinguished using pneumographic data [13].

### Body position and mandibular position

**Fig. 4:**
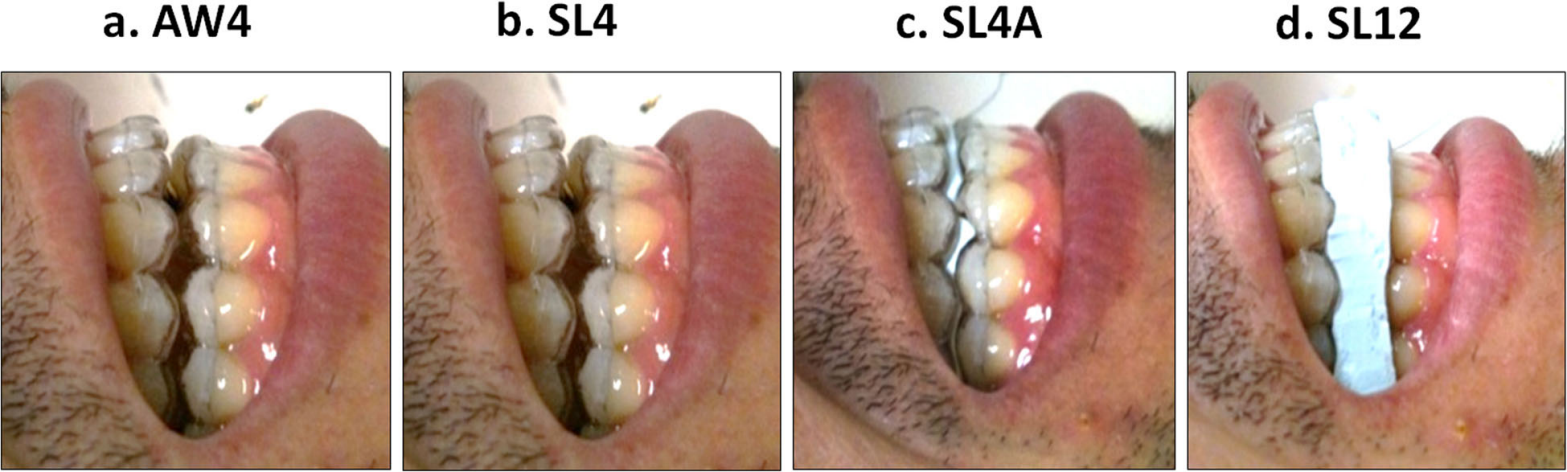
Photographs show the four test conditions evaluated in the present study. AW4: 4-mm BO and 0% protrusion while awake; SL4: 4-mm BO and 0% protrusion during sleep; SL4A: 4-mm BO and 50% protrusion during sleep; SL12: 12-mm BO and 0% protrusion during sleep

Four test conditions with specific mandibular positions were examined: 4 mm BO and 0% protrusion while awake (AW4), 4 mm BO and 0% protrusion during sleep (SL4), 4 mm BO and 50% protrusion during sleep (SL4A), and 12 mm BO and 0% protrusion during sleep (SL12) (Fig. 4). Each subject went to sleep in the supine position, with the dental chair reclined flat and the Frankfort plane maintained perpendicular to the floor.

### Recordings during sleep

Two to four test sessions were recorded for approximately 1 h until stage 2 sleep was observed on EEG. To set the mandibular position corresponding to each test condition, silicon bite blocks (LAB SILICONE®, SHOFU, Kyoto, Japan) were prepared before the recording sessions. The bite blocks were changed between sessions (Fig. 4).

### Statistical analysis

After tests for normality and equality of variance, statistical analysis of the data was performed with statistical software (SPSS ver. 21.0, IBM Corp., Armonk, NY, USA). The EMG activity of the masseter and GG muscles during maximum voluntary clenching and maximum tongue protrusion were used for standardized evaluations. The inspiration volume was standardized by the data obtained while the subject was in the upright AW4 position. Because normality assumptions may not be satisfied the Wilcoxon test was used for comparisons between the awake and sleeping conditions; *p* values of < 0.05 were deemed statistically significant. The Friedman test followed by post hoc Wilcoxon test with Bonferroni correction was used for comparisons among mandibular positions (SL4, SL4A, and SL12) in the sleeping condition; p values of < 0.05 were deemed statistically significant.

## Results

**Fig. 5:**
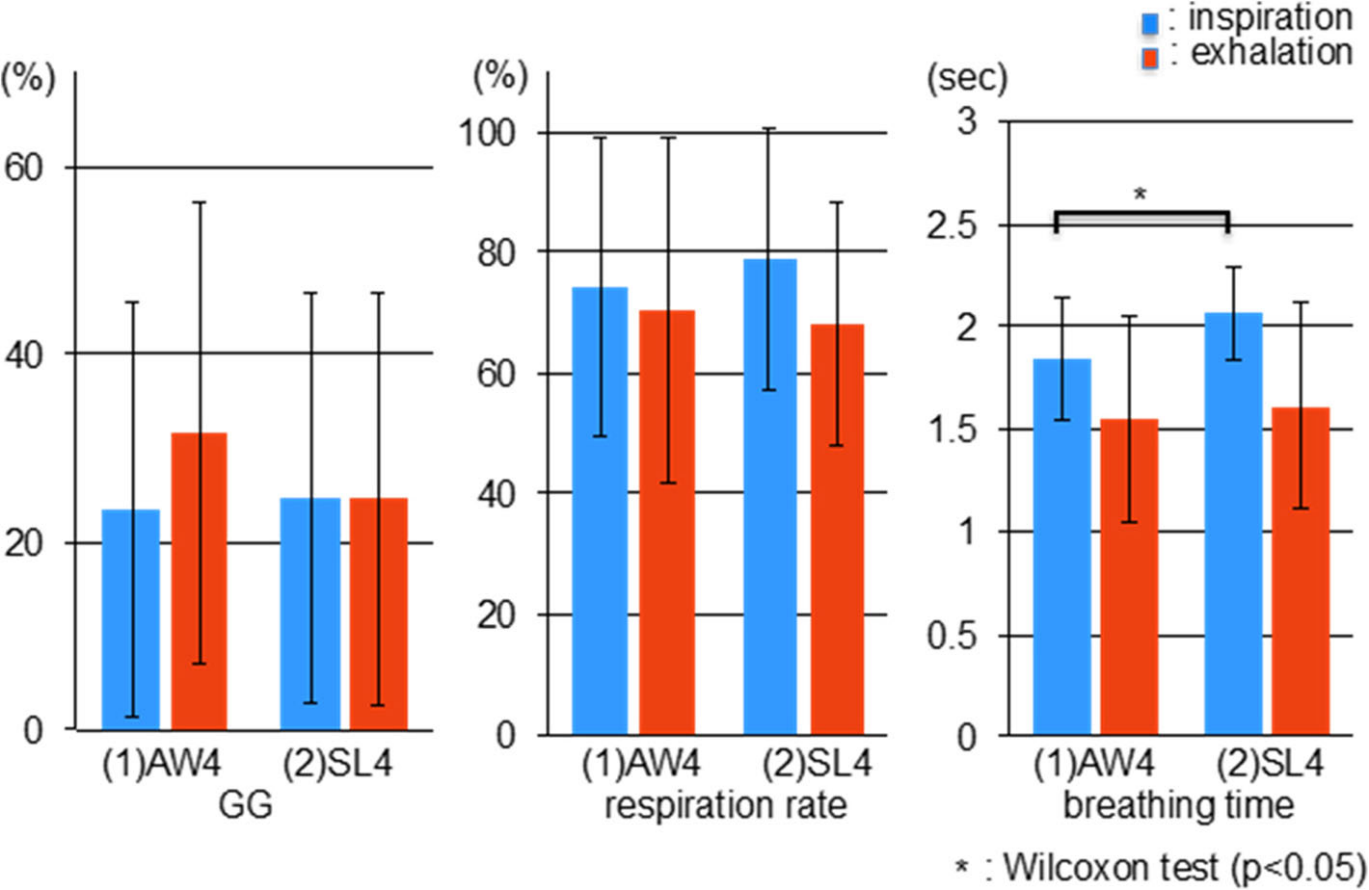
Comparison of the genioglossus (GG) muscle activity, respiration rate, and breathing time in the AW4 and SL4 test conditions. Only the breathing time for inspiration significantly differed between test conditions. AW4: 4 mm BO and 0% protrusion while awake; SL4: 4 mm BO and 0% protrusion during sleep

There were no significant differences in GG activity and respiration rate, while the breathing time of inspiration was significantly longer in SL4 compared with AW4 (*p* < 0.05), as shown in Fig. 5.

**Fig. 6:**
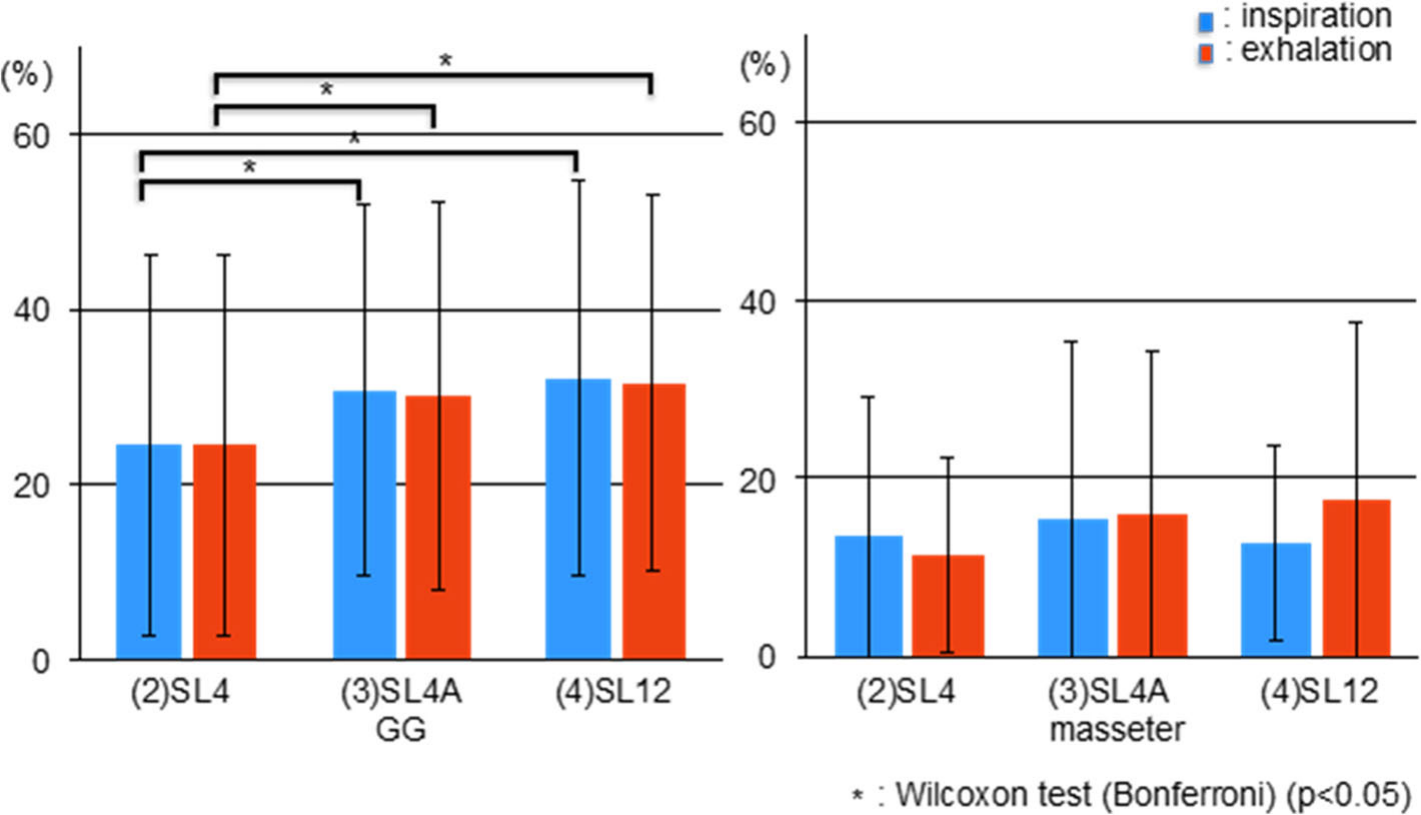
Comparisons of genioglossus (GG) and masseter activity in the three test conditions during sleep. SL4: 4-mm bite opening and 0% protrusion during sleep; SL4A: 4-mm bite opening and 50% protrusion during sleep; SL12: 12-mm bite opening and 0% protrusion during sleep

The GG activities in SL4A and SL12 were significantly higher than that in SL4 during both inspiration and exhalation (*p* < 0.05), as shown in Fig. 6. There were no significant differences in masseter activity between test conditions. One subject exhibited apparent masseter activity that continued for about 13 s during sleep.

**Fig. 7:**
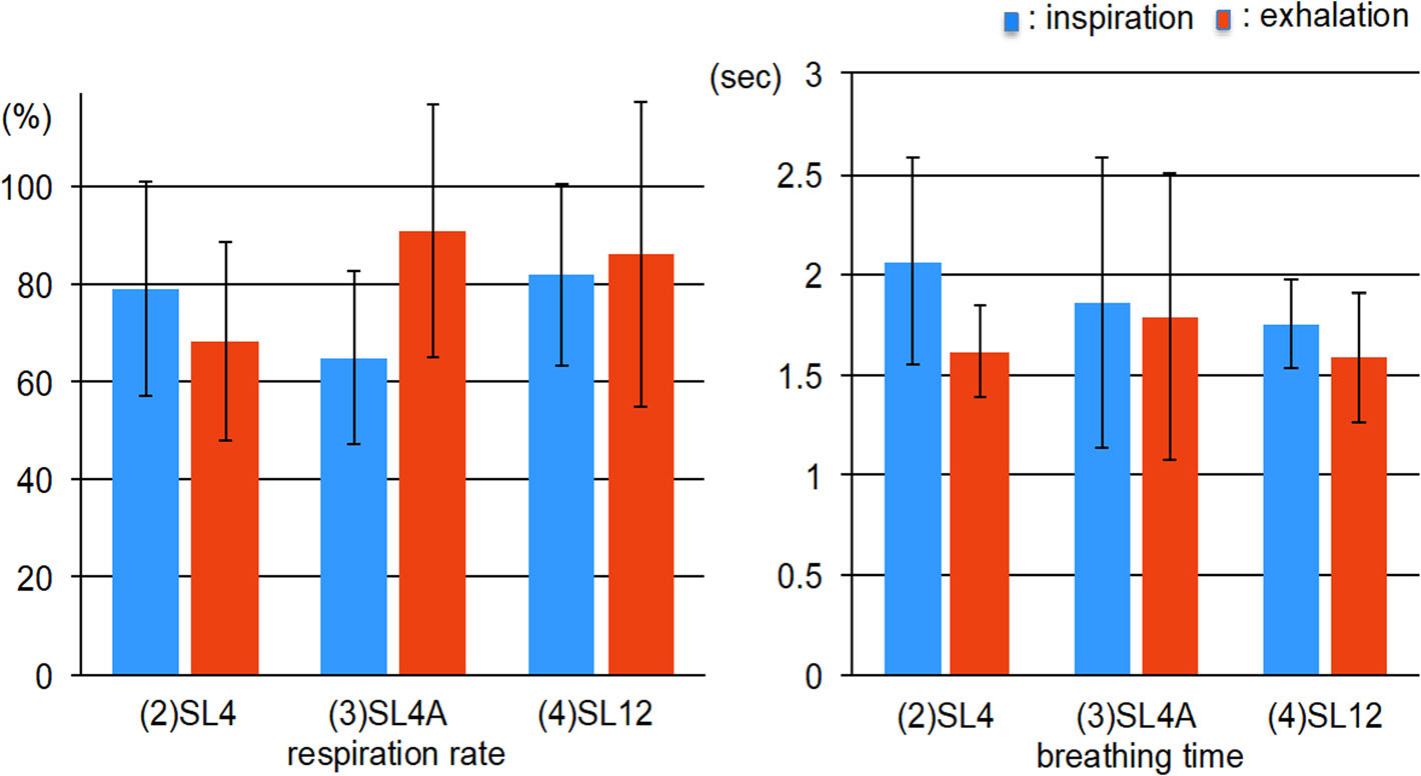
Comparisons of respiration rate and breathing time in the three test conditions during sleep. SL4: 4-mm bite opening and 0% protrusion during sleep; SL4A: 4-mm bite opening and 50% protrusion during sleep; SL12: 12-mm bite opening and 0% protrusion during sleep

There were no significant differences in respiration rate and breathing time in the three test conditions during inspiration or exhalation, as shown in Fig. 7.

## Discussion

The present study aimed to evaluate whether an increase in BO and mandibular anterior protrusion would promote an increase in GG and masseter activities, respiration rate, and breathing time while the subject was awake and asleep in the supine position. We found no significant differences in GG activity and respiration rate between the awake and asleep states; only breathing time when the subject was sleeping (SL4) was significantly increased compared with when the subject was awake (AW4). A previous study reported that normal subjects had small decrement GG activity during sleep compared with that while they were awake, while this state is inconsistent in OSAS patients, and GG activity shows large variable change during sleep [3]. The breathing time when the subject is asleep increases significantly and there were no significant differences between awake and sleep in GG activity and the respiration rate, this is not in accordance with a previous study that reported that people breathe more rapidly and shallowly while sleeping than when they are awake [17]. This might be due to the difference of experiment condition such as measurement timing and duration.

There were no significant differences in respiratory rate and breathing time between SL4, SL4A, and SL12. These results indicate that anterior protrusion and vertical displacement of the mandible do not affect the respiration in healthy subjects; this may be because there is no airway reduction in the supine position. A previous study also found no correlation between respiratory rate and body position in healthy subjects [18]. Further studies are needed to investigate the correlation between respiratory rate and body position in patients with OSAS.

There were no significant differences in masseter activity when the subjects had a BO of 4 mm, BO of 4 mm and 50% mandibular anterior protrusion, and BO of 12 mm during inspiration and exhalation. This suggests that BO and mandibular protrusion did not affect masseter muscle tension. However, the apparent masseter activity observed in one subject might have been an orofacial manifestation that often occurs during sleep; such nonspecific motor activation improves the configuration of the upper airway in association with another respiratory muscle in patients with OSAS [19]. In the present study, the level of GG activity in SL4A and SL12 was significantly higher than that in SL4 during both inspiration and exhalation; these results indicate that GG activity during sleep was influenced not only by the anterior protrusion of the mandible, but also by vertical mandibular displacement. The GG is one of the muscles responsible for upper airway dilation via the protrusion of the tongue body, and plays an important role in the physiological treatment of OSAS [20, 21]. It is still controversial whether an increased BO is needed in OAs. Previous studies reported that OSAS is more effectively treated with an increased BO; however, a minimal BO is more comfortable for the subject [6, 22]. Long et al. [13] reported that an increase in BO of 2–12 mm and even maximum mandibular protrusion may not negatively affect the temporomandibular joint. Therefore, in addition to mandibular protrusion, considering adjustment of the BO in OAs might minimize discomfort in patients with OSAS who are using OAs.

## Conclusion

The results of the present study led us to conclude that the GG muscle was influenced not only by the vertical mandibular position but also by its anterior displacement. To increase GG activity in patients with a small jaw opening (≤4 mm), the jaw should be protruded, a maneuver that is not necessary in those with a large opening (≥12 mm). Hence, both protrusion and the size of the mandibular opening should be examined and taken into account when evaluating the effectiveness of intraoral appliances for treating OSA.

## Data Availability

The dataset used and/or analyzed during the current study are available from the corresponding author on reasonable request.
